# Cross-cultural adaptation of the Amsterdam inventory for auditory disability and handicap to Brazilian Portuguese^☆^

**DOI:** 10.1016/j.bjorl.2018.07.011

**Published:** 2018-08-30

**Authors:** Sthella Zanchetta, Humberto Oliveira Simões, Pamela Papile Lunardelo, Marina de Oliveira Canavezi, Ana Cláudia Mirândola Barbosa Reis, Eduardo Tanaka Massuda

**Affiliations:** a Universidade de São Paulo (USP), Faculdade de Medicina de Ribeirão Preto, Departamento de Ciências da Saúde, São Paulo, SP, Brazil; b Universidade de São Paulo (USP), Faculdade de Medicina de Ribeirão Preto, Departamento de Neurociências e Ciências do Comportamento, São Paulo, SP, Brazil; c Universidade de São Paulo (USP), Faculdade de Ciências e Letras de Ribeirão Preto, Pós-graduação em Psicobiologia e Filosofia, São Paulo, SP, Brazil; d Universidade de São Paulo (USP), Faculdade de Medicina de Ribeirão Preto, Departamento de Oftalmologia, Otorrinolaringologia e Cirurgia de Cabeça e Pescoço, São Paulo, SP, Brazil

**Keywords:** Inquiries and questionnaires, Auditory perception, Reliability, Hearing loss, Adults, Investigações e questionários, Percepção auditiva, Confiabilidade, Perda de audição, Adultos

## Abstract

**Introduction:**

Patient-reported outcome measures, inventory and or questionnaire, allow patients to present their perspective of the impact of their individual condition on a day-to-day basis, independent of the analysis of test results by the expert clinician. Outcome measures are recommended when there is evidence showing their reliability, validity and sensitivity. There are standardized patient-reported outcome measures for hearing in English language; however, other languages lack these instruments.

**Objective:**

Adapt the Amsterdam inventory for auditory disability and handicap to Brazilian Portuguese and analyze its validation measures.

**Methods:**

We conducted two studies. In Study 1, we translated and adapted the Amsterdam inventory for auditory disability and handicap to Brazilian Portuguese according to good practice guidelines; this included the pre-test stage. In Study 2, we administered the Portuguese version to adults with and without hearing loss (*n* = 31 and 18, respectively) and analyzed the measures of instrument validation, reliability, and reproducibility. Moreover, we calculated the correlation between pure tone thresholds and scores on the questionnaire.

**Results:**

The results obtained in Study 1 demonstrated the feasibility of the translation process and the instrument's cultural adaptation, as well as its applicability, resulting in the Portuguese version of the Amsterdam inventory for auditory disability and handicap. In Study 2, the results revealed construct values for the questions and domains, as well as for the total reliable score. The intra-interviewer test–retest condition showed excellent reproducibility (ICC = 0.97). Finally, there was a strong positive correlation (*r* = 0.83) between the mean pure tone threshold and the hearing difficulties values, as measured by the instrument's scores.

**Conclusion:**

The English version of the Amsterdam inventory for auditory disability and handicap could be translated and adapted to Brazilian Portuguese. An analyses of the validation process yielded reliable, consistent, and stable results.

## Resumo

**Introdução:**

Medidas de resultados relatados pelo paciente, inventários e/ou questionários, permitem que os pacientes apresentem suas perspectivas do impacto de sua condição no dia a dia independentemente da análise dos resultados dos testes realizados pelo especialista. Esses instrumentos são recomendados quando há evidências que mostram sua confiabilidade, validade e sensibilidade. Existem medidas de resultados relatados pelo paciente padronizadas para a audição em língua inglesa; no entanto, esses instrumentos não existem em outras línguas.

**Objetivo:**

Adaptar o *Amsterdam inventory for auditory disability and handicap* para o português brasileiro e avaliar suas medidas de validação.

**Método:**

Realizamos dois estudos. No estudo 1, traduzimos e adaptamos o *Amsterdam inventory forauditory disability and handicap* para o português brasileiro de acordo com as diretrizes de boas práticas; incluindo a fase de pré-teste. No estudo 2, aplicamos a versão em português em adultos com e sem perda auditiva (n = 31 e 18, respectivamente) e analisamos as medidas de validação, confiabilidade e reprodutibilidade do instrumento. Além disso, calculamos a correlação entre os limiares de tons puros e os escores do questionário.

**Resultados:**

Os resultados obtidos no estudo 1 demonstraram a viabilidade do processo de tradução e adaptação cultural do instrumento, assim como sua aplicabilidade, proporcionaram a versão em português da *Amsterdam inventory for auditory disability and handicap*. No estudo 2, os resultados revelaram valores de constructo para as questões e domínios, bem como para o escore total confiável. A condição de teste-reteste intraentrevistador mostrou excelente reprodutibilidade (CCI = 0,97). Por fim, houve forte correlação positiva (r = 0,83) entre o limiar médio de tom puro e os valores das dificuldades auditivas, medidos pelos escores do instrumento.

**Conclusão:**

A versão em inglês do *Amsterdam inventory for auditory disability and handicap* foi traduzida e adaptada para o português brasileiro. Uma análise do processo de validação produziu resultados confiáveis, consistentes e estáveis.

## Introduction

Updated data from the World Health Organization (WHO) estimate that among the 360 million people with incapacitating hearing loss, 328 million are adults or elderly people.^1^ Pure-tone thresholds enable the identification of the magnitude of hearing loss, but they do not reflect the harmful impact of hearing loss on the daily life of the affected subject.^2^, ^3^, ^4^

The functional evaluation of hearing involves techniques and technological resources to identify hearing disorders, with hearing loss being the most frequently identified condition. This evaluation must be performed through the accurate analysis of signals obtained from a set of tests and/or exams, which is not complete without the elucidation of the hearing difficulties of the subject under evaluation. Patient-reported outcome measures (PROMs) allow patients to present their perspective of the impact of their condition on a day-to-day basis, independent of the analysis of test results by the expert clinician.^5^, ^6^, ^7^

Although PROMs are useful instruments that improve the quality of care offered to patients, they can be a potential trap for healthcare professionals with less knowledge about the requirements needed to select the instrument, as well as its application, analysis, and interpretation.^6^, ^7^ The use of PROMs is recommended when there is evidence showing their reliability, validity, sensitivity, and, finally, that the patient consents to the instrument (many subjects are fit to respond questions).^5^, ^6^, ^8^

There are standardized PROMs for hearing in English^8^; however, other languages lack these instruments.^9^ To deal with the latter situation, there are two possibilities for clinicians and researchers: (1) develop a new questionnaire and/or inventory or (2) translate and adapt instruments from another language.^6^, ^8^, ^10^ This option presents some advantages compared to the first one. If the instrument is translated and adapted as per directions,^8^, ^10^ it will enable comparative measures between different cultures and languages, and disseminate knowledge on the question investigated. For this action to occur, the “original” instrument must feature consistent, known measures of validity.

The Amsterdam Inventory for Auditory Disability and Handicap (AIADH) is one instrument to measure PROMs. Proposed by Kramer et al.,^11^ the instrument seeks to characterize auditory difficulties by abilities/stages and/or situations, such as in the presence or absence of noise. The results obtained using the instrument were significantly related to the hearing threshold level, as well as to the performance of speech perception tests, both in silence and noise.^2^ Subsequent studies have reported success in measuring self-reported hearing difficulties in the presence of hearing loss, including conductive hearing loss,^12^, ^13^, ^14^, ^15^ and its use has been extended to hearing screening,^16^ acting as a marker for occupational hearing health^17^, ^18^ and for evaluating patients with Auditory Processing Disorder.^18^, ^19^

The validation measures of the English-language AIADH as an instrument to measure self-perception of hearing difficulties in daily activities were detailed and studied by Meijer et al.^20^ According to the authors, the validity showed a significant correlation between the AIADH scores and tonal thresholds and a “highly satisfactory” reliability. The AIADH has already been translated into other languages, including Swedish,^15^ Cantonese,^21^ and Spanish,^22^ and their linguistic and cultural adaptations have shown instrumental validation measures similar to the original test. Another positive aspect of the AIADH was highlighted by Fuente et al.^21^, ^22^ According to them, auditory difficulties are collected into domains of investigation (detection, localization, discrimination/recognition, and intelligibility in silence and noise), which are in concordance with five auditory functions studied by the International Classification of Functioning, Disability and Health (ICF), proposed by the WHO^23^: detection, localization, lateralization, discrimination, and speech discrimination. All these characteristics make the AIADH an interesting instrument.

In view of the above factors, our objective was to translate and culturally adapt the AIADH into Brazilian Portuguese and analyze its results in order to legitimize its use. To fulfill these objectives, we performed two studies; the first verified the feasibility of translating and adapting the AIADH into Portuguese, and the second evaluated measures of reliability, validity, and acceptance of the instrument.

## Methods

A cross-sectional observational study was performed at the Medical School of Ribeirão Preto-University of São Paulo, with approval from the Research Ethics Committee of the Institution (n° 754.278/Ago 2014). All participants signed a informed consent form.

### Instrument

The AIADH, as proposed by Kramer et al.,^11^ is comprised of 30 questions. Of these, 28 questions are related to five fields of hearing: detection (questions 2, 10, 16, 22, and 28), localization (3, 9, 15, 21, and 27); discrimination/recognition (4, 5, 6, 17, 23, 24, 26, and 29), and intelligibility in silence (8, 11, 12, 14, and 20) and with noise (1, 7, 13, 19, and 25). For each question, there are four alternative answers, “almost never”, “sometimes”, “almost always”, and “always”, scored respectively with values of 3, 2, 1, and 0. The results are interpreted by evaluating the sum of the answers to all questions or by each of the five domains, producing a total and factor scores. A higher score indicates that the patient has greater hearing difficulty in situations dependent on the auditory sensory pathway. Two of the 30 questions – 18 and 30, are not related to the five auditory domains.^11^, ^12^ However, in this study, they were translated and adapted without having their measures taken, according to other studies on AIADH for other languages.^15^, ^21^, ^22^

### Study 1: Translation and validation of the AIADH into Brazilian Portuguese

#### Procedures

The process of translating and adapting the AIADH was performed in four stages according to recommendations in the literature,^9^ namely Study 1:

Stage 1 (Translation): The original version of the AIADH was delivered to three independent professionals, fluent in the English language and without prior knowledge of the instrument, who were asked to translate the instrument into Portuguese, resulting in three different versions called a, b, and c. The three versions were delivered to three consultants who were given guidelines for analyzing each question in the three versions and respond whether they had the same content. The answers of the consultants were subsequently analyzed by the authors of this work. Following satisfactory agreement between the authors for each question, a single version, referred to as version 1, of the AIADH in Portuguese was formulated.

Stage 2 (Retro-translation): The Portuguese version 1 of the AIADH was sent to a translator and retro-translated into English who had no prior knowledge of the instrument. The AIADH retro-translation and original English version were sent to two English-speaking consultants to see if the two versions could be characterized as possessing the same content and referred to the same instrument. The consultants’ answers were analyzed by examining the concordance between them. If this was satisfactory, version 1 of the AIADH in Portuguese was considered viable for study continuation; in the presence of a weak agreement, we would repeat Stage 1.

Stage 3 (Cultural adaptation): The version obtained after satisfactory agreement between the authors was then referred to a review committee to examine linguistic and cultural similarity. The test resulting from this step was referred to as adapted version 1.

Stage 4 (Pre-test): The adapted version 1 was tested in interviews regarding the intelligibility of its questions. We invited 25 randomly selected adults in a common area of the hospital linked to the university. The inclusion criteria were 18–50 years of age and patients who did not seek otorhinolaryngology and/or speech and hearing Services. All patients underwent pure-tone audiometry at the frequencies of 0.5, 1, 2, and 4 kHz. A mean value of >20 dB HL was interpreted as indicating hearing loss.^24^ Volunteers identified with hearing loss were referred for otorhinolaryngological evaluation and treatment.

All 25 volunteers, regardless of the mean tonal thresholds, were interviewed with the AIADH adapted version 1 and answered questions about the applicability of the inventory to verify whether additional instrument adaptations were required.^8^

The volunteers were instructed and asked about the following aspects: (a) Interrupting the interviewer in case a word they did not know or understand the meaning of emerged; (b) If the four possible answers to the questionnaire would meet their demands; and (c) If they would classify the questions as easy or hard to answer.

The material resulting from this stage, with satisfactory results’ concerning its applicability was designated the AIADH in Brazilian Portuguese (Pt-AIADH).

### Study 2: Validation of the Pt-AIADH

To analyze the instrument's validity and to determine whether it could be used to differentiate between populations with and without hearing loss, we recruited another 31 subjects. All subjects were previously identified with hearing loss by the public health system, and were referred to the Hearing Health Program of the Medical School of Ribeirão Preto-University of São Paulo.

The inclusion criteria were: a minimum age of 18 years, with no upper age limit; the presence of post-lingual acquired hearing loss of any nature; no previous experience of sound amplification devices; no apparent symptoms of conditions affecting communication observed during spontaneous conversation; and no need for caregivers and/or aids for locomotion and/or activities of daily living. This information was verified with a companion, when present. We did not establish exclusion criteria for this stage. The presence of hearing loss was identified when the mean frequencies of 0.5, 1, 2, and 4 kHz were >20 dB HL.^24^

The Pt-AIADH was performed by a single researcher on two days with the same subjects, with a minimum interval of 30 days between the interviews, to examine the test-retest reproducibility (reliability).

Thereafter, we compared the Pt-AIADH scores between subjects with and without hearing loss (Hearing Loss Group, HLG and Normal Hearing Group, NHG). The HLG was comprised of 31 volunteers from Study 2; the NHG was comprised of 18 of the 25 subjects from Study 1, Stage 4, who corresponded to those with mean tonal thresholds within normal limits for both ears.

### Statistical analyses

We used the Kappa concordance test in Stage 1 and 2 of Study 1.

In Study 2, the reliability of the Pt-AIADH was estimated using the Cronbach's alpha coefficient (*α*) test by question and domain. We used the Guttman Split-Half coefficient test^25^ to calculate the correlation between the two-part measures of the instrument (first 15 questions vs. last 15 questions). Reproducibility was determined by the degree of intraclass correlation within each domain (intraclass correlation coefficient) using an analysis of variance. The coefficient was estimated based on the mean squares obtained with the following formula: $r_{I}=\frac{\left(S_{b}^{2}-S_{w}^{2}\right)}{\left\{S_{b}^{2}+(n-1)S_{w}^{2}\right\}}$; in which $S_{b}^{2}$ is the mean square because of variation between classes, $S_{w}^{2}$ is the average square because of variation within classes, and n is the number of measurements within each class.

To verify whether the pure tone averages of the groups with and without hearing loss were different within and between the two groups for the different scores, we used the Mann–Whitney test. Finally, we estimated the correlation between values of the tonal means and total score using the Pearson's correlation test.

All analyses were performed with a 5% significance level and adjustments were performed using SAS Version 9.2 and R version 3.1; Prism 7 was used to produce graphs.

## Results

### Study 1

The analysis of the agreement among the three versions of the AIADH translated into Portuguese (Study 1, Stage 1), as well as between the original instrument and the resultant back-translation into Portuguese (Study 1, Stage 2) exhibited a level 1 agreement.

**Figure 1:**
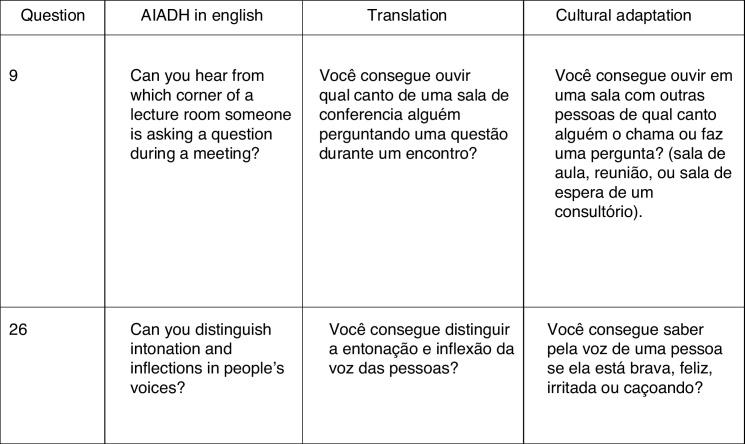
Example of two questions of the English version of AIADH that required cultural adaptation to Brazilian Portuguese.

The Portuguese version 1 of the AIADH was studied by the review committee for its grammatical and cultural adaptation (Study 1, Stage 3), which generated the adapted version 1. Fig. 1 presents two examples of the need for cultural adaptation.

The adapted version 1 was applied in a pre-test condition in 25 subjects with a mean age of 34 years (54.5% women). The calculation of the mean tonal thresholds suggested hearing loss in some subjects (Table 1). All 25 subjects answered the 30 questions; 100.00% of them said that there was no difficulty in answering or understanding the questions. Additionally, there were no requests to repeat any questions.

**Table 1:** Measures of hearing thresholds in dB HL (average across 0.5, 1, 2 and 4 kHz) for each study.

	Study 1		Study 2			
	Pretest (*n* = 25)		HLG (*n* = 31)		NHG (*n* = 18)	
	Better ear	Worse ear	Better ear	Worse ear	Better ear	Worse ear
Mean	23.4	27.8	49	67	18	19
Minimum	13	16	11	35	10	15
Maximum	62	68	97	120	20	20

HLG, Hearing Loss Group; NHG, Normal Hearing Group.

The described results enabled the identification the instrument as the Pt-AIADH (Appendix 1).

### Study 2: Validation of the Pt-AIADH

The Pt-AIADH was applied to 31 subjects, with a minimum age of 25 and a maximum of 92 years (mean = 59 years; SD = 21.4). All subjects presented with hearing loss, thus constituting the group with Hearing Loss (HLG), with 74.2% (23/31) and 25.8% (8/31) being of the sensorineural and mixed types, respectively. Concerning laterality, 96.8% (30/31) had bilateral impairment and only 3.2% (1/31) demonstrated unilaterality. The descriptive values of the tonal means are shown in Table 1.

### Validation of the Pt-AIADH

The Pt-AIADH responses were studied in relation to their reliability. Internal consistency was the first variable examined. We initially performed the calculation for each of the 30 questions separately, then for each of the domains, and later the instrument. Next, the correlation between the two halves of the questionnaire was calculated. For these analyses, the lowest value was always ≥0.80, except for the result of the analysis of the second part of the inventory, which was 0.72 (Table 2).

**Table 2:** Measures of reliability for each inventory questions were categorized for the five auditory factors of the Pt-AIADH from Study 2, in subjects with hearing loss.

Subjective hearing factor/question	Cronbach alpha		Guttman split half
Sound detection		0.887644	
2	0.886675		
10	0.887153		
16	0.888909		
22	0.886159		
28	0.883654		
Sound localization		0.907495	
3	0.889503		
9	0.884981		
15	0.889916		
21	0.890397		
27	0.888343		
Sound discrimination		0.880698	
4	0.888936		
5	0.884541		
6	0.885177		
17	0.886531		
23	0.888680		
24	0.885830		
26	0.884741		
29	0.883654		
Speech perception in quiet		0.893755	
8	0.886408		
11	0.888820		
12	0.884254		
14	0.882869		
20	0.881357		
Speech perception in noise		0.884200	
1	0.882992		
7	0.883439		
13	0.889445		
19	0.888398		
25	0.887890		
Total score		0.942207	
Coefficient total			0.87
Alpha for part 1			0.80
Alpha for part 2			0.72

Table 3 shows the reliability measures obtained from the present version as well as the other measures already taken from AIADH to facilitate visualization and comparison.

**Table 3:** Comparative illustration of AIADH measures of reliability and their different cross-cultural adaptations.

	AIADH				
	Original	Swedish	Cantonese	Spanish	Portuguese
*Cronbach alpha value*					
Inventory	–	–	0.96	0.97	0.94
Sound detection	0.77	0.77	0.85	0.84	0.88
Sound localization	0.88	0.88	0.89	0.87	0.90
Sound discrimination	0.89	0.89	0.91	0.89	0.88
Speech perception in quiet	0.85	0.85	0.86	0.83	0.89
Speech perception in noise	0.81	0.81	0.90	0.84	0.88

*Guttman split half*					
Total	–	–	–	0.97	0.87
Part 1	–	–	–	0.94	0.82
Part 2	–	–	–	0.94	0.72

Original, Kramer et al., 1995; Swedish, Hallberg et al., 2008; Cantonese, Fuente et al., 2010; Spanish, Fuente et al., 2012; Portuguese, Zanchetta et al., present study.

The second variable analyzed for reliability was the intrasubject reproducibility (i.e., test/retest reliability); 100.00% of the 31 subjects with hearing loss responded to the Pt-AIADH at two time points, the score results at the two points were similar, if not equal (Table 4). The interval between the first and second interview ranged from 30 to 55 days (mean = 40 days; SD = 10). The intraclass correlation between the test and retest was estimated and yielded the following results: detection = 0.94 (min = 0.90, max = 0.98); localization = 0.99 (min = 0.99, max = 0.99); discrimination = 0.91 (min = 0.86, max = 0.97); speech intelligibility in quiet = 0.98 (min = 0.97, max = 0.99); speech intelligibility with noise = 0.98 (min = 0.97, max = 0.99); and total score = 0.97 (min = 0.96, max = 0.99).

**Table 4:** Test and retest scores and standard deviation (SD) for auditory factors of Pt-AIADH.

Measures	Interview	Auditory factors					Total score
		Sound detection	Sound localization	Sound discrimination	Speech perception in quiet	Speech perception in noise	
Mean	1st	9.29/2.8	10.10/2.3	11.97/5.4	9.61/2.9	11.87/2.6	52.77/12.8
	2nd	9.16/2.8	10.0/2.4	11.68/5.49	9.6/2.95	11.94/2.4	53.42/13.1
Median	1st	9.0	11.0	12.0	10	12.0	54.0
	2nd	9.0	11.0	12.0	10	12.0	53.0
Quartile 1	1st	7.0	8.0	7.0	8.0	11.0	44.0
	2nd	7.0	8.0	8.0	8.0	11.0	44.0
Quartile 3	1st	12.0	12.0	16.0	12.0	13.0	61.0
	2nd	12.0	12.0	16.0	12.0	13.0	62.0
Minimum–Maximum	1st	4.0–14.0	5.0–13.0	3.0–23.0	3.0–15	5.0–20.0	22.0–70.0
	2nd	4.0–14.0	4.0–14.0	3.0–23.0	3.0–15	5.0–20.0	20.0–80.0

Correlations among the five domains of Pt-AIADH were also calculated (Table 5). The results suggest that there is a moderate correlation among the domains, with only one exception, found between the auditory location and speech perception in silence.

**Table 5:** Correlation between auditory factors of Pt-AIADH.

Factors Pt-AIADH	Sound discrimination	Sound localization	Speech perception in noise	Speech perception in quiet
Sound localization	0.50			
Speech perception in noise	0.61	0.50		
Speech perception in quiet	0.55	0.31	0.52	
Sound detection	0.53	0.43	0.56	0.61

Subsequently, we conducted a study to verify whether the instrument differentiated groups with and without hearing loss (HLG and NHG, respectively). The HLG comprised 31 subjects from Study 2, while the NHG comprised 18 (72.00%) of the 25 subjects in Study 1, Stage 4 of the pre-test phase, who corresponded to those subjects with mean tonal thresholds within normal limits.

Initially, we analyzed how different the tonal means were between the two groups. The HLG exhibited values of tonal means of the best and worst ear inferior to those of the NHG (mean of the best ear HLG vs. NHG, *p* < 0.0001*; *U*′ = 32; mean of the worst ear HLG vs. NHG, *p* < 0.0001*; *U*′ = 0) (Table 1).

**Figure 2:**
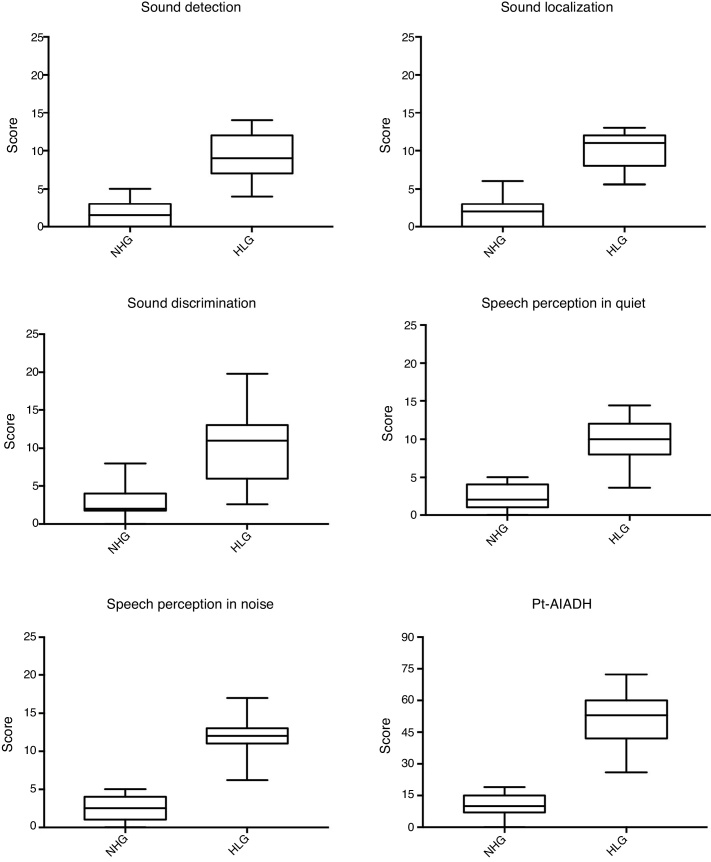
Mean values scores for each of the auditory factors, as total, of the Pt-AIADH in Normal Hearing Group (NHG) and Hearing Loss Group (HLG).

**Figure 3:**
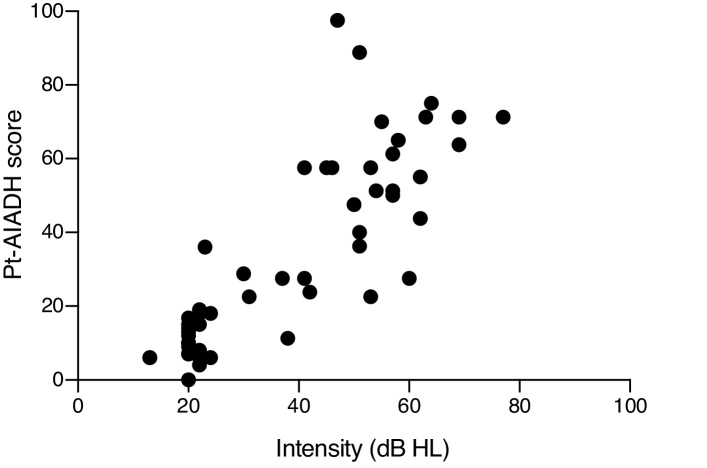
Means of the pure-tone (across 0.5, 1, 2 and 4 kHz of the better ear) and total score obtained in both groups, with and without hearing loss (*n* = 49).

Next, the results of the total Pt-AIADH scores and of each of its domains (detection, localization, discrimination/recognition, and intelligibility with and without noise) were compared between the two groups. For all domains, significant differences (*p* < 0.0001*) were observed, showing that the HLG presented greater hearing difficulty than did the NHG (Fig. 2). Finally, we identified the correlation between the tonal mean values of the subjects (with and without hearing loss, *n* = 49) with the total Pt-AIADH score. We used the corresponding tonal means of the best ears of the two groups and found a strong significantly positive correlation (*r* = 0.8303, *p* < 0.001*, 95% CI 0.7207–0.8994); briefly, the higher the tonal mean, the greater the hearing difficulty (Fig. 3).

## Discussion

### Study 1

The analysis of the agreement between judges concerning the three independent translations, as well as the back-translation, was selected to produce greater reliability, as recommended by the specialized literature.^10^ The degrees of agreement obtained at this stage were the largest possible, allowing the Portuguese version 1 of the AIADH to move to the grammatical and cultural adaptation stage, generating an adapted version 1, an essential step because it allows certain phenomena, such as self-reported hearing loss in this study, to be identified in different cultures.^6^, ^8^, ^10^ With the adapted version, we evaluated the first of four validation criteria, instrument acceptance.^6^, ^7^ In the pre-test stage, none of the volunteers reported that they did not understand any of the 30 questions, which allowed the Pt-AIADH version to be finalized, suggesting that it is a feasible instrument.

### Study 2

The Pt-AIADH was established in an interview conducted with 31 subjects with hearing loss, with the objective of evaluating their reliability in terms of internal consistency and reproducibility, criteria two and three of the validation process.^6^

The AIADH is a multidimensional instrument because there are five factors,^2^ so therefore, it is necessary to evaluate all the factors and not only the total score.^6^

The isolated results that we obtained from the Pt-AIADH suggest that it contains sufficient reliability for its use, because the literature indicates that *α* = 0.70 is a suitable cut-off, with *α* = 0.90 considered excellent^25^, ^26^; our values here ranged from 0.72 to 0.94. A limiting factor in the results presented in this study is the absence of a factor analysis, as carried out in the original study and in the Swedish version.^15^ The referred analysis would enable to identify whether the AIADH questions would present the same underlying structure,^27^ nonetheless, the Pt-AIADH sample number did not enable its calculation. This also occurred for the other versions, which did not carry out the same analysis factor. On the other hand, although a factor analysis is lacking, the absence does not undermine the results obtained from the measurements of the other construct variables, which are quite satisfactory.^25^, ^26^

When we compared these results with translations of the AIADH into Swedish, Cantonese, and Spanish, it was possible to verify that the reliability values are similar, and that they may be interpreted as satisfactory results.^15^, ^21^, ^22^

All measures of the intra-observer reproducibility of Pt-AIADH showed favorable results concerning the stability of the instrument, as well as similarity to other versions in other languages.^15^, ^21^, ^22^

The potential for translating and culturally adapting PROMs from English to Brazilian Portuguese, while maintaining validation measures of the respective instruments, is precedented.^28^, ^29^, ^30^ Castro et al.^28^ examined an inventory for subjects with dizziness, the Dizziness Handicap Inventory, while Mondelli et al.^29^ examined the Satisfaction with Amplification in Daily Life applied in 30 subjects with hearing loss who used acoustic amplification. In both cases, the authors translated and adapted the respective instrument; however, they evaluated only one aspect of reliability, reproducibility. However, in the translation and adaptation of Beliefs and Attitudes on Hearing Loss Preventions to Brazilian Portuguese, Bramatti et al.^30^ evaluated more validation questions, such as the construct, and the results yielded values that give confidence to use the respective instrument.

The use of Pt-AIADH in subjects with and without hearing loss yielded distinct results between them, that is, it could distinguish the condition for which it was constructed. The higher the averaged pure tone thresholds, the greater the self-reported hearing difficulty, consistent with studies of the AIADH translated and adapted into other languages.^15^, ^21^, ^22^ These measures are important because clinical tests for auditory measurement do not encompass daily activities in which the subject is involved.

It is important to emphasize that no Portuguese PROM aimed at examining hearing, focusing on the hearing difficulty, with known validation variables presented, has been developed. This makes the Pt-AIADH the first reliable instrument for its purpose, available in Brazilian Portuguese. Lastly, as a final consideration, we emphasize that future studies should characterize domains according to audiometric characteristics, as well as the sensitivity and specificity of the instrument.

## Conclusion

The AIADH is an instrument that could be translated and adapted to Brazilian Portuguese, with the translated version referred to as the Pt-AIADH. The validation process analyses showed reliable, consistent, and stable results, as with versions in other languages, including the original one.

## Funding

This work was supported by 10.13039/501100003593CNPq (grant: 148046/2013-4).

## Conflicts of interest

The authors declare no conflicts of interest.

## Figures and Tables

**Table:** 

1.	Você consegue entender um vendedor quando está em uma loja lotada?
	[Can you understand a sales assistant's speech when you are in a crowded store?]
2.	Você consegue acompanhar uma conversa com uma pessoa em um lugar silencioso?
	[Can you keep up with a conversation with a person in a quiet place?]
3.	Na rua, ao ouvir um carro se aproximando, você consegue saber imediatamente de qual lado ele está vindo?
	[On the street, when you hear a car approaching, can you immediately recognize from which direction it is coming?]
4.	Você consegue ouvir os carros passando na rua?
	[Can you hear cars passing by?]
5.	Você reconhece pessoas de sua família pela voz delas?
	[Can you recognize people from your family by their voice?]
6.	Você consegue reconhecer a melodia em música ou canções?
	[Can you recognize melody in music or songs?]
7.	Você consegue acompanhar uma conversa em um lugar lotado/cheio?
	[Can you follow a conversation in a busy/crowded place?]
8.	Você consegue conversar ao telefone em um lugar silencioso?
	[Can you talk on the phone in a quiet place?]
9.	Você consegue ouvir em uma sala com outras pessoas de qual canto alguém o chama ou faz uma pergunta? (sala de aula, reunião ou sala de espera de um consultório).
	[When someone asks a question during a meeting, can you hear from which corner of a conference room it is coming?]
10.	Você consegue ouvir alguém se aproximando por trás?
	[Can you hear someone approaching from behind?]
11.	Você reconhece um apresentador de TV pela voz?
	[Can you recognize a TV presenter by his/her voice?]
12.	Você consegue entender a letra de uma música que está sendo cantada?
	[Can you understand the lyrics of a song when it is being sung?]
13.	Você consegue facilmente acompanhar uma conversa com alguém em um carro ou ônibus?
	[Can you easily keep up with a conversation with someone in a car or bus?]
14.	Você consegue entender um apresentador de jornal na televisão?
	[Can you understand a news presenter on TV?]
15.	Você imediatamente olha para o lado certo quando alguém o chama na rua?
	[Do you immediately look toward the correct direction when someone calls you on the street?]
16.	Você consegue ouvir os barulhos da casa como a descarga do banheiro, a máquina de lavar roupa, a água?
	[Can you hear house noises, such as toilet flushing, washing machine whirring, and water flowing?]
17.	Você consegue diferenciar o som de um ônibus e de um carro?
	[Can you differentiate the sound of a bus from a car?]
18.	Você já teve a experiência de uma música, ou outro som, estar muito alto, enquanto as outras pessoas que estão junto com você não referem a mesma sensação?
	[Have you experienced the feeling of listening to music that is too loud for you but not for other people?]
19.	Você consegue acompanhar a conversa, entre poucas pessoas, durante um jantar?
	[Can you follow the conversation between a few people during a meal?]
20.	Você consegue entender um apresentador no rádio?
	[Can you understand a newsreader on the radio?]
21.	Em uma casa silenciosa, você consegue ouvir de qual canto alguém está falando com você?
	[When someone is talking to you in a quiet house, can you hear from which corner his/her voice comes?]
22.	Você consegue ouvir quando alguém bate palma, ou toca a campainha ou o interfone da sua casa?
	[Can you hear the doorbell?]
23.	Você consegue distinguir, na maioria das vezes, as vozes femininas e masculinas?
	[Can you differentiate between the voice of a man and a woman?]
24.	Você consegue ouvir o ritmo de uma música ou de canções?
	[Can you follow the rhythm of music or songs?]
25.	Você consegue acompanhar uma conversa com alguém em uma rua movimentada?
	[Can you keep up with a conversation with someone on a busy street?]
26.	Você consegue distinguir pela voz, se uma pessoa está brava, irritada ou está caçoando?
	[Can you differentiate by voice if someone is angry, irritated, or joking?]
27.	Ao ouvir uma buzina de carro, você consegue saber de qual direção ela vem vindo?
	[Can you hear from which direction the sound of a car horn is coming?]
28.	Você ouve os pássaros cantando lá fora?
	[Can you hear birds chirping outside?]
29.	Quando ouve uma música, você consegue reconhecer e distinguir os instrumentos musicais?
	[When you listen to music, can you recognize and distinguish the musical instruments used?]
30.	Você perde partes da música enquanto escuta músicas ou canções?
	[Do you feel that parts of music are missing when listening to music or sounds/songs?]

## References

[1] World Health Organization. Geneve: Deafness and hearing loss – fact sheet/detail. Available from: http://www.who.int/mediacentre/factsheets/fs300/en/ [cited 22.05.18].

[2] Giolas T. (1983). The self-assessment approach in audiology: state of the art. Audiology.

[3] Newman C.W., Weinstein B.E., Jacobson G.P., Hug G.A. (1990). The Hearing Handicap Inventory for Adults: psychometric adequacy and audiometric correlates. Ear Hear.

[4] Musiek F.E., Shinn J., Chermak G.D., Bamiou D.E. (2017). Perspectives on the pure-tone audiogram. J Am Acad Audiol.

[5] Dawson J., Doll H., Fitzpatrick R., Jenkinson C., Carr A.J. (2010). The routine use of patient reported outcome measures in healthcare settings. BMJ.

[6] Brédart A., Marrel A., Abetz-Webb L., Lasch K., Acquadro C. (2014). Interviewing to develop Patient-Reported Outcome (PRO) measures for clinical research: eliciting patients’ experience. Health Qual Life Outcomes.

[7] Nelson E.C., Eftimovska E., Lind C., Hager A., Wasson J.H., Lindblad S. (2015). Patient reported outcome measures in practice. BMJ.

[8] Hall D.A., Zaragoza Domingo S., Hamdache L.Z., Manchaiah V., Thammaiah S., Evans C. (2018). International Collegium of Rehabilitative Audiology and TINnitusResearch NETwork. A good practice guide for translating and adapting hearing-related questionnaires for different languages and cultures. Int J Audiol.

[9] Diao M., Sun J., Jiang T., Tian F., Jia Z., Liu Y. (2014). Comparison between self-reported hearing and measured hearing thresholds of the elderly in China. Ear Hear.

[10] Guillemin F., Bombardier C., Beaton D. (1993). Cross-cultural adaptation of health-related quality of life measures: literature review and proposed guidelines. J Clin Epidemiol.

[11] Kramer S.E., Kapteyn T.S., Festen J.M., Tobi H. (1995). Factors in subjective hearing disability. Audiology.

[12] Kramer S.E., Kapteyn T.S., Festen J.M., Tobi H. (1996). The relationships between self-reported hearing disability and measures of auditory disability. Audiology.

[13] Kramer S.E., Kapteyn T.S., Festen J.M. (1998). The self-reported handicapping effect of hearing disabilities. Audiology.

[14] Korsten-Meijer A.G., Wit H.P., Albers F.W. (2006). Evaluation of the relation between audiometric and psychometric measures of hearing after tympanoplasty. Eur Arch Otorhinolaryngol.

[15] Hallberg L.R., Hallberg U., Kramer S.E. (2008). Self-reported hearing difficulties, communication strategies and psychological general well-being (quality of life) in patients with acquired hearing impairment. Disabil Rehabil.

[16] Molander P., Nordqvist P., Oberg M., Lunner T., Lyxell B., Andersson G. (2013). Internet-based hearing screening using speech-in-noise: validation and comparisons of self-reported hearing problems, quality of life and phonological representation. BMJ.

[17] Pawlaczyk-Łuszczyńska M., Dudarewicz A., Zamojska M., Sliwinska-Kowalska M. (2012). Self-assessment of hearing status and risk of noise-induced hearing loss in workers in a rolling stock plant. Int J Occup Saf Ergon.

[18] Fuente A., McPherson Y.B., Hormazabal X. (2013). Self-reported hearing performance in workers exposed to solvents. Rev Saude Publica.

[19] Bamiou D.E., Iliadou V.V., Zanchetta S., Spyridakou C. (2015). What can we learn about auditory processing from adult hearing questionnaires?. J Am Acad Audiol.

[20] Meijer A.G., Wit H.P., TenVergert E.M., Albers F.W., Muller K.J.E. (2003). Reliability and validity of the (modified) Amsterdam Inventory for Auditory Disability and Handicap. Int J Audiol.

[21] Fuente A., McPherson B., Kwok E.T.T., Chan K., Kramer S.E. (2012). Adaptation of the Amsterdam Inventory for Auditory Disability and Handicap into Cantonese. Aust New Zeal J Audiol.

[22] Fuente A., McPherson B., Kramer S.E., Hormazábal X., Hickson L. (2012). Adaptation of the Amsterdam Inventory for Auditory Disability and Handicap into Spanish. Disabil Rehabil.

[23] World Health Organization. Geneve: The International Classification of Functioning, Disability and Health (ICF). Available from: http://www.who.int/classifications/icf/en/ [cited 22.05.18].

[24] Bureau International D’Audiophonologie. Belgium: Audiometric Classification of Hearing Impairment; BIAP Recommendation 02/1. Available from: https://www.biap.org/en/recommandations/recommendations/tc-02-classification/213-rec-02-1-en-audiometric-classification-of-hearing-impairments/file [cited 22.05.18].

[25] Feldt L.S., Charter R.A. (2003). Estimating the reliability of a test split into two parts of equal or unequal length. Psychol Methods.

[26] Nunnaly J.C. (1978).

[27] Hair J.F., Black W.C., Babin B.J., Anderson R.E., Tatham R.L. (2006).

[28] Castro A.S.O., Gazzola J.M., Natour J., Ganança F.F. (2007). Brazilian version of the Dizziness Handicap Inventory. Pro Fono.

[29] Mondelli M.F., Magalhães F.F., Lauris J.R. (2011). Cultural adaptation of the SADL (satisfaction with amplification in daily life) questionnaire for Brazilian Portuguese. Braz J Otorhinolaryngol.

[30] Bramatti L., Morata T.C., Marques J.M., Martini U.G. (2012). Translation and adaptation of the questionnaire “beliefs and attitudes on hearing loss prevention” into Brazilian Portuguese. Rev CEFAC.

